# Handbook on Obsessive-Compulsive and Related Disorders

**DOI:** 10.1192/pb.bp.115.052803

**Published:** 2017-08

**Authors:** Lynne M. Drummond

This multi-author book examines the disorders categorised in DSM-5 as obsessive-compulsive and related disorders (OCaRDs) and also covers two other related disorders: illness anxiety and obsessive-compulsive personality disorders. The text is written by experts in the field, many of whom were instrumental in developing the concept of OCaRDs in DSM-5. Unlike many multi-author publications, this is brief and to the point. Each of the chapters is arranged in a structured format which includes a general introduction; diagnostic criteria and symptomatology; epidemiology; comorbidity; course and prognosis; psychosocial impairment; developmental considerations; gender-related issues; cultural aspects of phenomenology; assessment and differential diagnosis; aetiology and pathophysiology; treatment (somatic as well as cognitive and behavioural) and a summary of key points at the end. Most chapters also contain illustrative case vignettes which demonstrate the disorders and their potential severity.

This excellent title should be on the bookshelf of every psychiatrist, whether working with adults or children. Mental health workers, managers and commissioners often overlook common conditions such as obsessive-compulsive disorder and body dysmorphic disorder, regarding them as less severe and important than conditions such as schizophrenia. This work describes the hugely detrimental effects these conditions can have on the individual's mortality, morbidity and social functioning. It also considers newly defined disorders, such as hoarding and skin-picking disorders. These conditions are poorly understood and have generally not been researched extensively. They do, however, appear to be widespread and often have extreme consequences on the individual's mental and physical well-being. For example, hoarding disorder, which was previously often classified as obsessive-compulsive disorder or else obsessive-compulsive personality disorder, may affect up to 6% of the adult population. Excessive hoarding can lead to death due to fire risk or the falling of large numbers of possessions resulting in an avalanche. It also frequently coexists with self-neglect and – owing to the extreme shame – social isolation. Nevertheless, few community mental health teams in the UK offer comprehensive treatment for hoarding disorders. Some enlightened councils have developed hoarding protocols incorporating housing agencies, social services, and mental and physical health services but overall these are patchy and rare.

Hopefully, heightened awareness will lead to systematic research and a better understanding of the disorders and their treatment, as well as the development of effective services. This handbook would be a perfect introduction to these areas for a busy practitioner seeking brief but authoritative information.

